# Influence of Connector Design on Displacement and Micromotion in Tooth-Implant Fixed Partial Dentures Using Different Lengths and Diameters: A Three-Dimensional Finite Element Study

**DOI:** 10.3390/ma17174416

**Published:** 2024-09-07

**Authors:** Hisham A. Mously, Ghada H. Naguib, Abou Bakr Hossam Hashem, Ahmed O. Abougazia, Abdulelah M. Binmahfooz, Mohamed T. Hamed

**Affiliations:** 1 Department of Oral and Maxillofacial Prosthodontics, Faculty of Dentistry, King Abdulaziz University, Jeddah 21589, Saudi Arabia; dr.mously@gmail.com (H.A.M.); abinmahfooz@kau.edu.sa (A.M.B.); mthamed@kau.edu.sa (M.T.H.); 2 Department of Restorative Dentistry, Faculty of Dentistry, King Abdulaziz University, Jeddah 21589, Saudi Arabia; 3 Department of Oral Biology, School of Dentistry, Cairo University, Cairo 12613, Egypt; 4 Dental Department, Research Institute of Ophthalmology, Giza 11261, Egypt; aboubakrhashem@yahoo.com; 5 Private Practice, Giza 12573 Egypt; ahmedgaziaa@gmail.com; 6 Department of Fixed Prosthodontics, School of Dentistry, Cairo University, Cairo 12613, Egypt

**Keywords:** tooth-implant fixed partial dentures (FPDs), connector design, displacement, micromotion, periodontal ligament (PDL), finite element analysis, rigid connectors, non-rigid connectors, biomechanical performance

## Abstract

The literature presents insufficient data evaluating the displacement and micromotion effects resulting from the combined use of tooth-implant connections in fixed partial dentures. Analyzing the biomechanical behavior of tooth-implant fixed partial denture (FPD) prothesis is vital for achieving an optimum design and successful clinical implementation. The objective of this study was to determine the relative significance of connector design on the displacement and micromotion of tooth-implant-supported fixed dental prostheses under occlusal vertical loading. A unilateral Kennedy class I mandibular model was created using a 3D reconstruction from CT scan data. Eight simulated designs of tooth-implant fixed partial dentures (FPDs) were split into two groups: Group A with rigid connectors and Group B with non-rigid connectors. The models were subjected to a uniform vertical load of 100 N. Displacement, strain, and stress were computed using finite element analysis. The materials were defined as isotropic, homogeneous, and exhibiting linear elastic properties. This study focused on assessing the maximum displacement in various components, including the bridge, mandible, dentin, cementum, periodontal ligament (PDL), and implant. Displacement values were predominantly higher in Group B (non-rigid) compared to Group A (rigid) in all measured components of the tooth-implant FPDs. Accordingly, a statistically significant difference was observed between the two groups at the FPD bridge (*p* value = 0.021 *), mandible (*p* value = 0.021 *), dentin (*p* value = 0.043 *), cementum (*p* value = 0.043 *), and PDL (*p* value = 0.043 *). Meanwhile, there was an insignificant increase in displacement values recorded in the distal implant (*p* value = 0.083). This study highlighted the importance of connector design in the overall stability and performance of the prosthesis. Notably, the 4.7 mm × 10 mm implant in Group B showed a displacement nearly 92 times higher than its rigid counterpart in Group A. Overall, the 5.7 mm × 10 mm combination of implant length and diameter showcased the best performance in both groups. The findings demonstrate that wider implants with a proportional length offer greater resistance to displacement forces. In addition, the use of rigid connection design provides superior biomechanical performance in tooth-implant fixed partial dentures and reduces the risk of micromotion with its associated complications such as ligament overstretching and implant overload, achieving predictable prognosis and enhancing the stability of the protheses.

## 1. Introduction

Historically, dental implants have been effectively utilized in treating fully edentulous patients [1,2], with more recent trends showing their increased application in treating partially edentulous patients as well [3]. The success of implant treatments, including those for partially edentulous patients, is well documented, exhibiting favorable survival rates for both the implants and the supported prostheses [4]. For instance, one systematic review reported implant survival rates between 92% and 97% over a minimum of five years [5]. Another review focused on the longevity of implant-supported prostheses, showing a survival rate of 96.4% at five years and 93.9% after ten years [6]. However, despite these high success rates, both biological and technical complications are not uncommon, affecting up to 33.6% of cases within five years [6]. Although titanium implants are biocompatible, less frequent complications have been reported, such as titanium allergies [7] and corrosion [8]. Titanium allergies affect approximately 0.6% of the population, which result from the body’s reaction to titanium components, potentially due to ion absorption through the skin or mucosal contact, or from implant corrosion processes [7]. Another limitation of titanium implants is their dark color, which can pose an esthetic issue, particularly in patients with a thin gingival phenotype. The gray shadow of the titanium may show through the peri-implant mucosa, negatively affecting the esthetic outcome [9,10,11]. To address these drawbacks, zirconia implants were introduced. These implants have been in use in Europe since the early 2000s and have seen increased usage in the USA following FDA approval in 2011 [12]. Additionally, zirconia’s opaque white color, combined with its biocompatibility, ability to osseointegrate, and low tendency to attract bacterial plaque, makes it an excellent material choice for dental implants [13]. However, their main disadvantage is aging under high compressive stresses with the subsequent formation of microcracks, which render them the procedure of choice, particularly in the esthetic zone [14,15]. Accordingly, titanium implants remain the standard material for replacing missing posterior teeth [16].

In certain clinical scenarios, connecting osseointegrated implants to natural teeth might be unavoidable. This is particularly common in Kennedy Class I or II cases, where a three-unit bridge links an implant to the terminal natural tooth. Such an approach can negate the need for additional implants distal to the last natural tooth, which can be both costly and complicated by local factors such as limited bone volume or the presence of vital structures that impede implant placement. By avoiding complex bone grafting procedures, this method can reduce patient morbidity and the likelihood of complications [17,18].

Two systematic reviews investigated the survival rate of tooth-implant fixed partial dentures (FPDs). Their findings reported an estimated survival rate of 90.8% after 5 years and 82.5% after 10 years [19]. Additionally, the authors emphasized that while implant-implant FPDs are generally the first choice of treatment, tooth-implant FPDs remain an accepted treatment option with predictable success rate, given they are clinically justified [20]. Numerous studies have investigated the mechanisms of occlusal load transmission from the surface of a prosthesis to the underlying bone, considering that the biological reaction of the bone around the implant can significantly affect bone remodeling, the integration of the implant, and the longevity of the prosthesis. Despite these insights, the specific stress, strain, and micro-displacement responses of dental implants under typical occlusal loading conditions remain poorly understood because these responses cannot be directly measured in vivo [21,22,23].

Analyzing the biomechanical behavior of tooth-implant fixed partial denture (FPD) prothesis is vital for achieving an optimum design and successful clinical implementation. The proper management of loading on the alveolar bone and adjacent structures is essential, as insufficient load can lead to bone resorption and potential implant loosening. Conversely, excessively high stresses and displacements can cause bone loss and microfractures [24,25,26].

Three-dimensional (3D) finite element analysis is extensively utilized in the dental industry as a robust method for analyzing the complex biomechanics of implants and the surrounding bone tissue [27,28]. This technique offers a detailed view of how displacement, stress, and strain affect the tooth-implant fixed partial denture and implant denture systems [29]. By accounting for the heterogeneous properties of biomaterials and their complex geometries, 3D finite element analysis enables a comprehensive assessment of these dental structures [30,31,32]. Tsouknidas et al. [33] were among the early researchers to explore the effects of displacement and stress distribution in teeth connected to implants. Their study highlighted the importance of bone quality and its critical role as a biomechanical parameter affecting both displacement and stress distribution within the tooth-implant hybrid prothesis.

Several other studies have displayed various factors that can influence the displacement and micromotion of dental implants. These factors include implant thread design [34], surface roughness [35], implant microgeometry [36,37], abutment angulation [38], cortical bone thickness [39], bone density [40], and the direction of the load applied [41,42].

The main concern with tooth-implant connections is the differential tooth movement within the periodontal ligament (PDL), as compared to osseointegrated implants within bone. This difference in mobility led to a dispute over which type of connector design to use in protheses connecting teeth to osseointegrated dental implants [43,44,45].

Accordingly, in a recent finite element stress analysis study by Naguib et al. [46], their results recommended the use of a rigid connector type in the construction of tooth-implant-supported fixed dental prostheses. However, the literature presents insufficient data evaluating the displacement and micromotion effects resulting from the combined use of tooth-implant connections in fixed partial dentures. This study’s hypothesis supports the use of rigid connection in tooth-implant fixed partial protheses for enhanced biomechanical performance and stability. Therefore, the objective of this investigation was to determine the relative significance of the connection design in the displacement and micromotion of tooth-implant-supported fixed dental prothesis under occlusal vertical loading.

## 2. Materials and Methods

This investigation adhered to the TRIPOD guidelines (Transparent reporting of a multivariable prediction model for Individual Prognosis or Diagnosis) [47]. A unilateral Kennedy class I mandibular model was created using a 3D reconstruction from CT scan data utilizing Mimics 8.1 software. The CT scans were acquired in DICOM format with a Toshiba multi-slice CT scanner, following the manufacturer’s recommended settings: 140 kV, 130 mA, a resolution of 512 × 512 pixels, a slice thickness of 0.33 mm, with no tilt towards the maxilla and a slight 19-degree tilt of the mandible to prevent overlap of the maxillary teeth over the mandibular teeth. The scans were initially set in the axial plane for the anterior–posterior orientation and adjusted for superior and inferior views, then processed with the Mimics 8.1 software (Materialise Interactive Medical Image Control System—Mimics 8.1 for Intel ×86 Pentium III + V8.5.0.23, Leuven, Belgium).

The region of interest (ROI) was defined based on radiopacity levels, selecting bone, teeth, and prosthetic components within a Hounsfield range of 578–3071. Distinct masks with varying colors were applied to differentiate the teeth from the mandibular structure using the regional growing feature; a separate mask was also generated for the teeth to delineate them from the mandible. The missing teeth were digitally reconstructed, and a dental implant was positioned in the second molar site while considering the second premolar (enhanced with simulated cementum and periodontal ligament) as the abutment. A Ni-Cr alloy served as the material for the metal framework of the 3-unit FDP, linking the dental implant at the distal end to the natural second premolar tooth.

**Figure 1:**
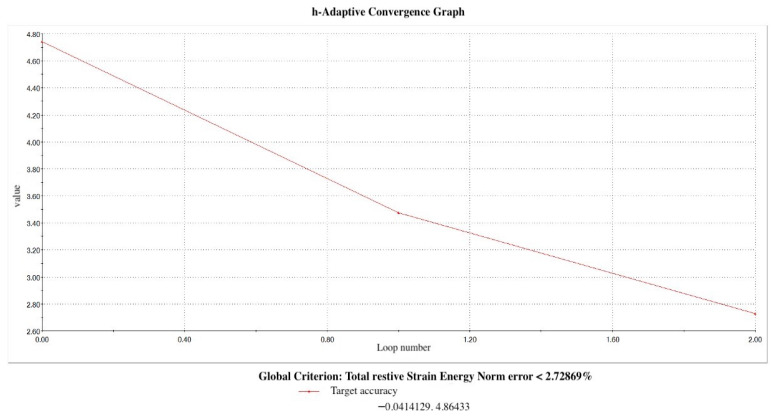
H-Adaptive convergence graph.

**Figure 2:**
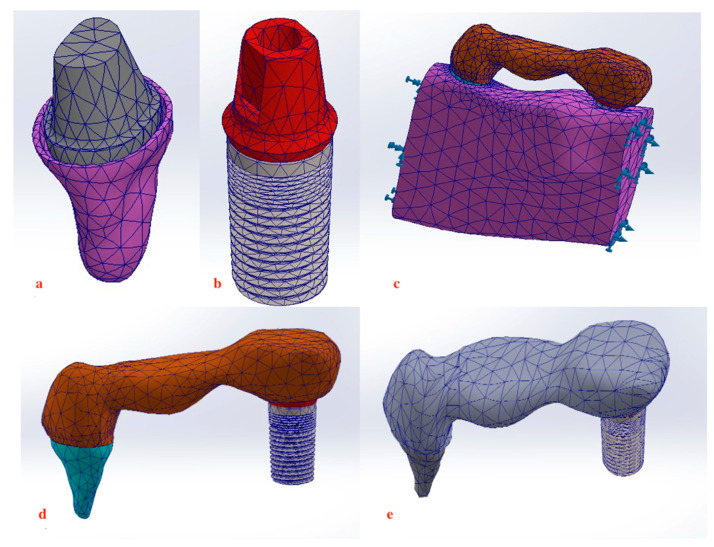
Meshing of structures. (**a**,**b**) 3D final model of tooth abutment and distal implant. (**c**) Assembly of 3-unit metal framework on the abutments embedded within bone. (**d**,**e**) Assembly of 3-unit metal framework connecting the distal implant to second premolar with rigid and non-rigid connection.

Dimensional assessments were performed using the software’s measuring tool, aiding in the development of a 3D model that facilitated the mapping and transitioning of the mesh to the Rapidform XOS system version 3.0 (mesh to solid software) for constructing a parametric model complete with an engineering drawing and a finite element analysis plan (Solidworks Premium 64 Edition, Waltham, MA, USA). In addition, automatic adjustments were implemented by the system to ensure a precision level under 3% (Figure 1) (Figure 2a–e). The elastic modulus value and Poisson’s ratio corresponding to the physical properties of materials used to model different structures is displayed in Table 1. Mesh optimization was conducted to refine this study’s outcomes; the mesh was adjusted to closely replicate the anatomical structures studied, ensuring that larger elements did not compromise reliability, while smaller elements remained within acceptable tolerances. 

A Screw-Plant titanium implant system was utilized and designed in accordance with specifications provided by Implant Direct LLC, based in Malibu Hills, CA, USA. These implants are distinguished by their spiral, self-tapping form and conical shape, featuring miniature threads measuring between 2 and 2.5 mm. They also possess double lead threads extending to the implant’s apex and are equipped with an internal hex platform that is 2 mm in length. Eight simulated designs of tooth-implant FDPs were split into two groups. The used root form implants had lengths of 10, 11.5, and 13 mm and diameters of 3.7, 4.7, and 5.7 mm. The main difference between groups was the connector type. Group A tooth-implant FDPs had a mesial and distal rigid connector. Group B tooth-implant FDPs had a mesial non-rigid connector in their design between the pontic and tooth.

Fixed partial denture (FPD) models with rigid connections were simulated as a single entity and set to feature bonded interfaces where no relative movement between adjacent surfaces was permitted. Conversely, FPD models with non-rigid connections were designed to allow no penetration; this setup permitted separation and relative movement between interfaces with a friction coefficient of 0.5 and a constant vertical separation of 4 mm, chosen for its ability to accurately simulate the resistance to sliding and separation for the non-rigid connector, reflecting realistic biomechanical behavior in tooth-implant fixed partial dentures under occlusal loading [53,54,55]. The width of the periodontal ligament (PDL) space was consistently set at 0.2 mm [56]. Materials across all simulations are biocompatible and were defined as isotropic, homogeneous, and exhibiting linear elastic properties [57,58,59]. The analytical framework applied a load or sequence of loads on the structures, with calculations performed using systems of algebraic equations. The results were subsequently presented in terms of displacement, strain, and stress at various structural nodes.

**Figure 3:**
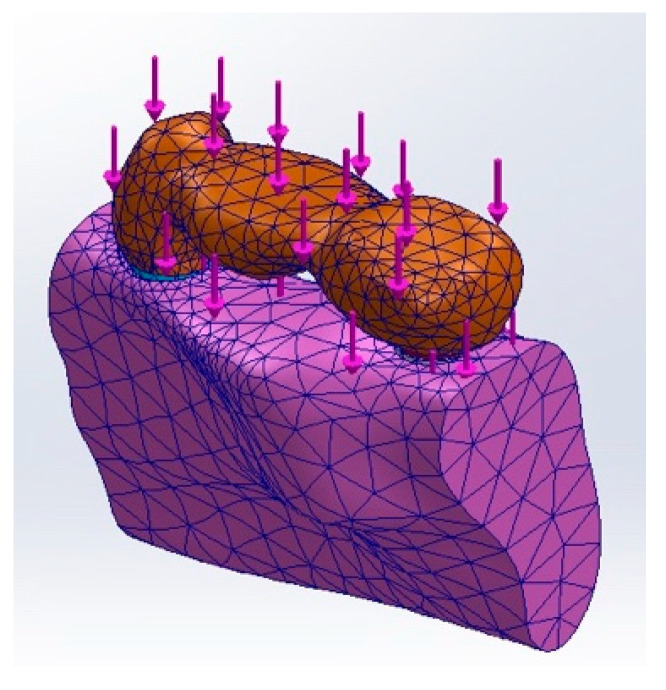
Application of load.

In the analysis, each of the six models was subjected to a uniform vertical load of 100 N [60]. Various studies [61,62,63,64] have demonstrated that a 100 N load lies within the normal range of biting forces. Accordingly, load was applied to the three-unit bridge covering the central fossae of the first premolar, molar, and second molar (Figure 3). The displacement at each component was computed under each load condition, examining different anatomical parts including the FDP, surrounding bone, distal implant bone, cementum, PDL, and implant itself. To enhance the visual clarity and interpretation of displacements, the palette was expanded to include a broader range of colors, with the maximum displacement depicted in a gradient from blue (minimum displacement) to red (maximum displacement). This approach allows for a clear visualization of displacement fields, assisting in the identification of potential mechanical issues or areas requiring design optimization.

## 3. Results

This study evaluated the biomechanical performance of tooth-implant fixed partial dentures (FPDs) with different connector designs. Two groups were analyzed: tooth-implant FPD with rigid connection (Group A) and tooth-implant FPD with rigid connection with non-rigid connection (Group B). This study focused on assessing the maximum displacement (millimeter) in various components (bridge, mandible, dentin, cementum, PDL, implant) under a uniform vertical load of 100 N.

**Figure 4:**
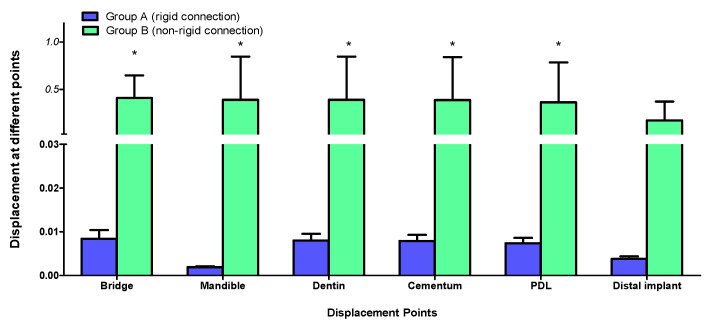
Graph representing measured displacement values in Group A (rigid) and Group B (non-rigid) at different regions. * Indicates statistical significance (* *p* < 0.05) between Group A (rigid connection) and Group B (non-rigid connection), as determined by the Mann–Whitney test. Data were expressed as mean ± standard deviation.

**Figure 5:**
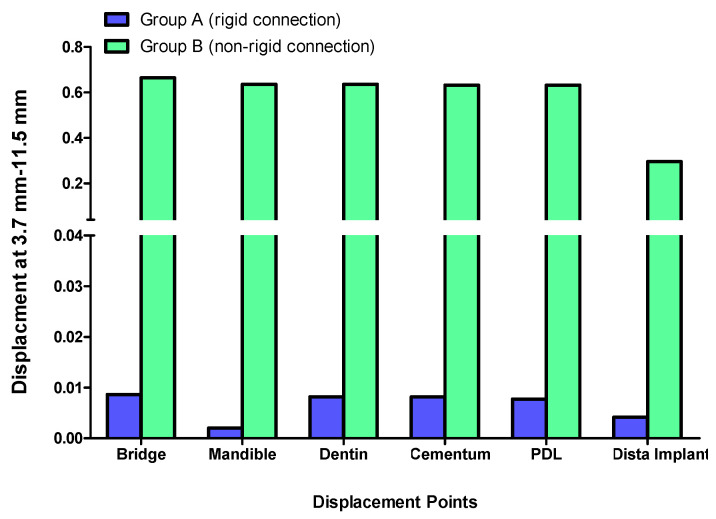
Displacement analysis between Group A (rigid) and Group B (non-rigid) at different regions for 3.7–11.5 mm implant length and diameter.

**Figure 6:**
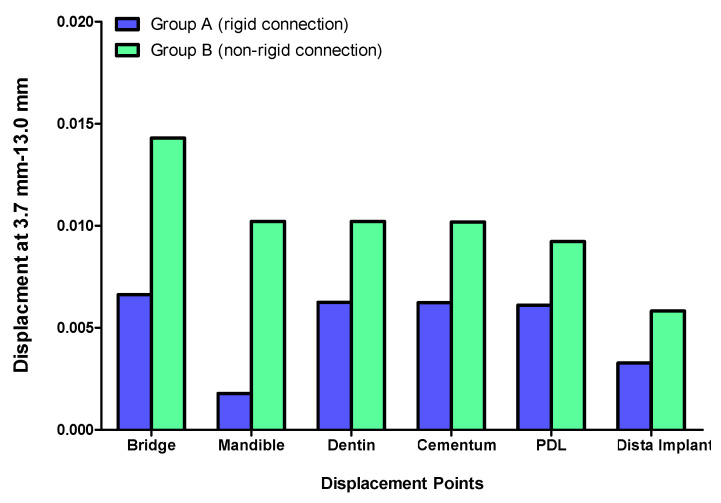
Displacement analysis between Group A (rigid) and Group B (non-rigid) at different regions for 3.7–13 mm implant length and diameter.

**Figure 7:**
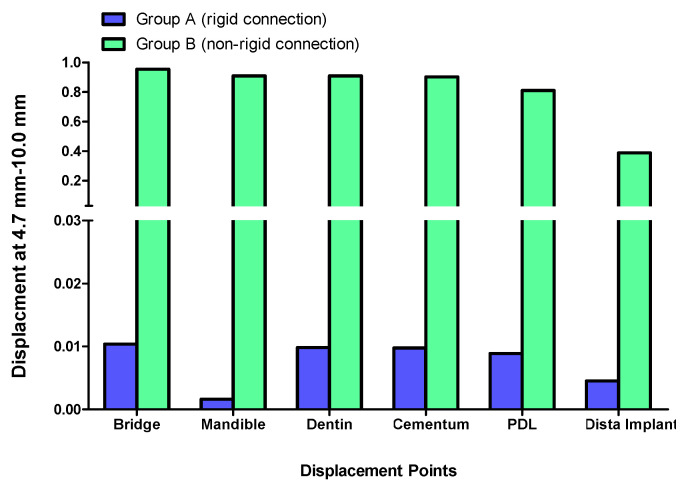
Displacement analysis between Group A (rigid) and Group B (non-rigid) at different regions for 4.7–10 mm implant length and diameter.

**Figure 8:**
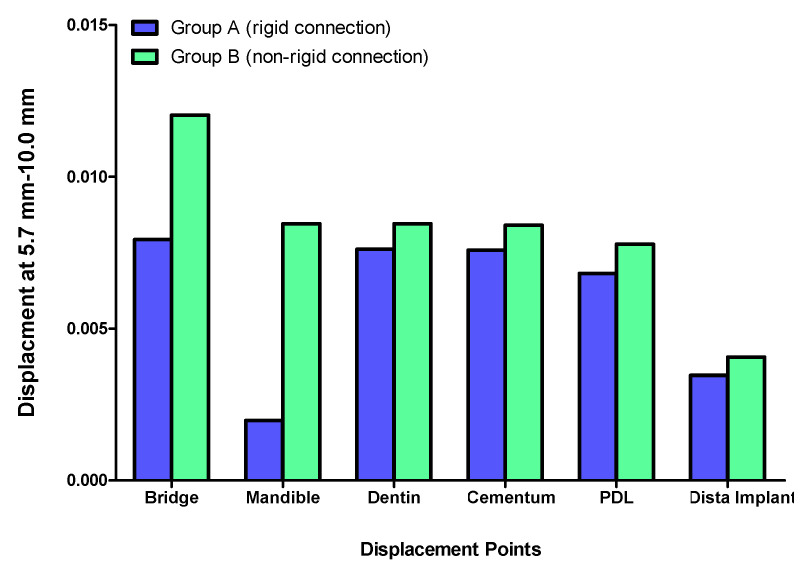
Displacement analysis between Group A (rigid) and Group B (non-rigid) at different regions for 5.7–10 mm implant length and diameter.

**Figure 9:**
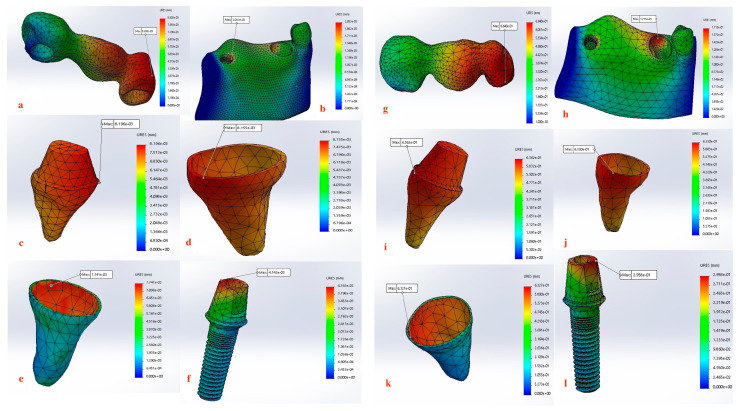
(**a**–**l**) Maximum Displacement values calculated at each component in Group A with rigid connection (**a**–**f**) vs. Group B with non-rigid connection (**g**–**l**) (3.7 mm × 11.5 mm).

**Figure 10:**
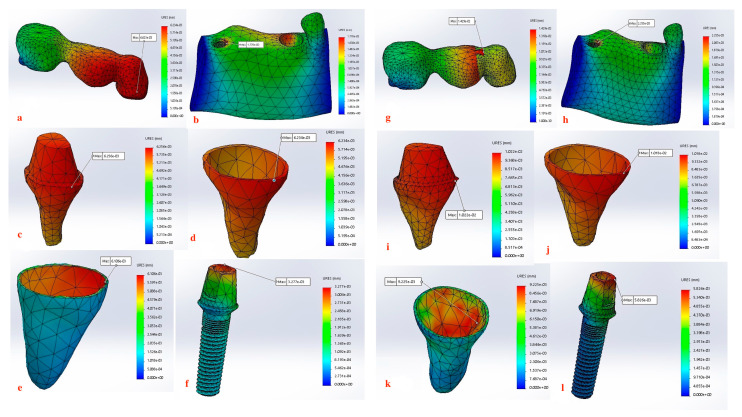
(**a**–**l**) Maximum Displacement values calculated at each component in Group A with rigid connection (**a**–**f**) vs. Group B with non-rigid connection (**g**–**l**) (3.7 mm × 13 mm).

**Figure 11:**
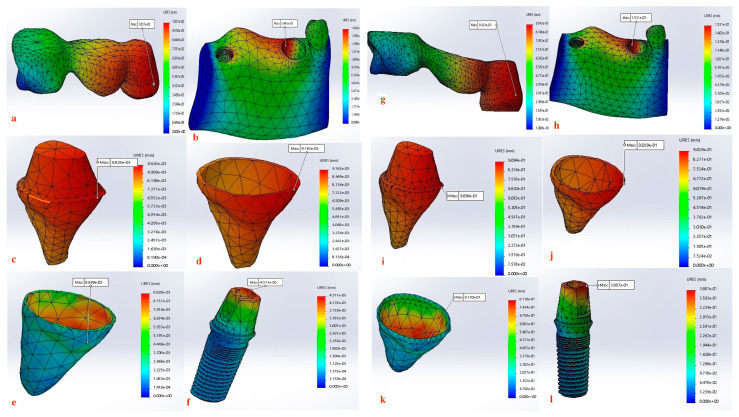
(**a**–**l**) Maximum Displacement values calculated at each component in Group A with rigid connection (**a**–**f**) vs. Group B with non-rigid connection (**g**–**l**) (4.7 mm × 10 mm).

**Figure 12:**
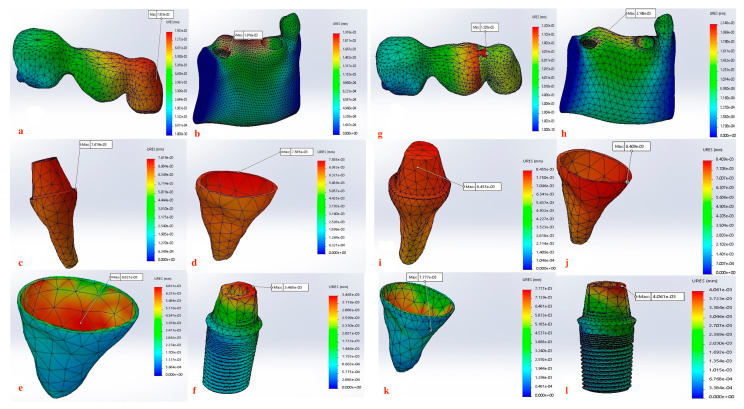
(**a**–**l**) Maximum Displacement values calculated at each component in Group A with rigid connection (**a**–**f**) vs. Group B with non-rigid connection (**g**–**l**) (5.7 mm × 10 mm).

Displacement values were predominantly higher in Group B (non-rigid) compared to Group A (rigid). Accordingly, displacement values were collected across different combinations of implant length and diameter at specific measurement points (e.g., FPD bridge, mandible, dentin, cementum, PDL, and implant). These values were then averaged, and the mean ± standard deviation was calculated for each group using the Mann–Whitney U test. A statistically significant difference was observed between the two groups at bridge (*p* value = 0.021 *), mandible (*p* value = 0.021 *), dentin (*p* value = 0.043 *), cementum (*p* value = 0.043 *), and PDL (*p* value = 0.043 *). Meanwhile, an insignificant increase in displacement values was recorded in the distal implant (*p* value = 0.083) (Table 2 and Table 3, Figure 4, Figure 5, Figure 6, Figure 7, Figure 8, Figure 9a–l, Figure 10a–l, Figure 11a–l and Figure 12a–l).

### 3.1. Displacement at the FPD Bridge

The displacement at the FPD bridge was consistently higher in Group B (non-rigid connectors) compared to Group A (rigid connectors) across all implant dimensions. The maximum displacement observed was 0.9543000 mm for the 4.7 mm diameter and 10 mm length implant in Group B, marking approximately a 92-fold difference than the corresponding rigid design (0.0103700 mm) in Group A (Figure 7).

### 3.2. Displacement at the Mandible

The displacement at the mandible was uniformly higher in Group B (non-rigid connectors) compared to Group A (rigid connectors) across all implant dimensions. The maximum displacement observed was 0.17150 mm for the 3.7 mm diameter and 11.5 mm length implant in Group B, which was 82.66-fold higher than the corresponding rigid design in Group A (0.00205) (Figure 5).

### 3.3. Displacement at the Dentin

Group B exhibited significantly higher dentin displacement compared to Group A. The most notable difference was observed for the 4.7 mm diameter and 10 mm length implant, where Group B showed a displacement of 0.90940 mm, which was 92.4-fold higher than Group A (0.00983 mm) (Figure 7).

### 3.4. Displacement at the Cementum

The cementum displacement was substantially higher in Group B compared to Group A. For the 4.7 mm diameter and 10 mm length implant, Group B showed a cementum displacement of 0.90290 mm, which was 92.3-fold higher than Group A (0.00976 mm) (Figure 7).

### 3.5. Displacement at the PDL

The PDL displacement was also significantly higher in Group B compared to Group A. The 4.7 mm diameter and 10 mm length implant displayed the highest difference in displacement, where Group B showed a PDL displacement of 0.81100 mm, which was 91.1-fold higher than Group A (0.00890 mm) (Figure 7).

### 3.6. Displacement at the Implant

The implant displacement was generally higher in Group A (rigid connectors) compared to Group B (non-rigid connectors). The maximum implant displacement was observed for the 4.7 mm diameter and 10 mm length implant in Group B (0.388700 mm), which was 86-fold higher than the corresponding non-rigid design (0.0045110 mm) in Group A (Figure 7).

### 3.7. Influence of Implant Length and Diameter

**Figure 13:**
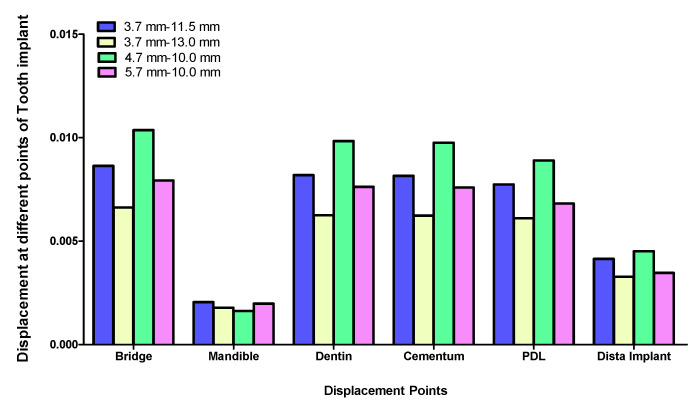
Effect of implant diameter and length on displacement in tooth-implant FPD measured at different regions in Group A (rigid connection).

**Figure 14:**
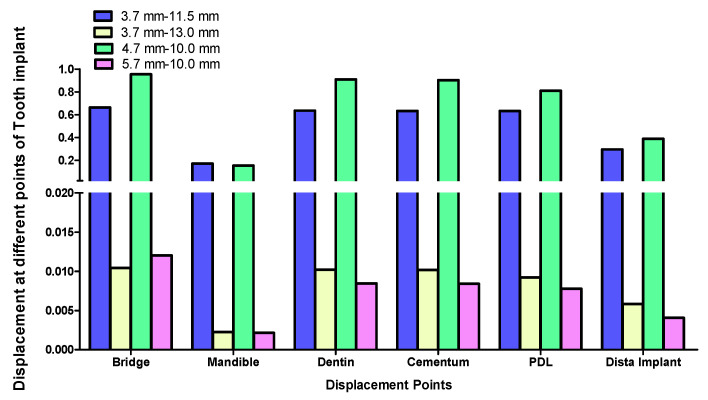
Effect of implant diameter and length on displacement in tooth-implant FPD measured at different regions in Group B (non-rigid connection).

The data suggest a negative correlation between implant length and displacement, where shorter implants tend to have lower displacement values. In terms of diameter, wider implants also exhibit lower displacement, indicating better stability. Among the combinations studied, the 5.7 mm × 10 mm in the rigid connector group showcases the best performance, exhibiting the least displacement across most measured components (Table 4, Figure 13 and Figure 14).

## 4. Discussion

The objective of this study was to broaden the understanding of the tooth-implant fixed partial protheses biomechanics. Following Nagiub et al.’s [46] stress analysis on such a hybrid prothesis where rigid connections provided optimal stress distribution over non-rigid connections, the results of this study augment the current knowledge on the effect of different connection designs in relation to displacement and micromotion. Our findings demonstrated significantly higher displacement values in Group B (non-rigid connectors) compared to Group A (rigid connectors) across the measured regions of the FPD bridge, mandible, dentin, cementum, and PDL, while there was an insignificant increase in displacement in the distal implant (Table 3), thus prominently underlining the importance of connector design in the overall stability and performance of the prosthesis. This was consistent with previous studies that have shown non-rigid connectors to result in sub-optimal biomechanical behavior in combined tooth-implant fixed partial dentures [46,65,66,67].

The rigid connector design (Group A) consistently exhibited lower displacements compared to the non-rigid connector design (Group B). This was especially evident in all measured components within the tooth-implant FPD (Table 2, Figure 4). The 4.7 mm × 10 mm combination of implant length and diameter in Group B (non-rigid) displayed substantially higher displacements than their corresponding values in Group A (rigid) (Figure 7). In Group B (non-rigid), the dentin had a displacement value of 0.90940 mm, a staggering 92.4-fold increase over Group A’s 0.00983 mm (rigid). Similarly, cementum in Group B displayed a 92.3-fold higher displacement (0.90290 mm) compared to Group A (0.00976 mm), while PDL region in Group B showed a maximum displacement of 0.81100 mm, which was 91.1-fold higher than Group A (0.00890 mm). Finally, displacement recorded at implant showcased an 86-fold higher displacement value (0.388700) than its corresponding rigid design in Group A (0.0045110). This marked increase in displacement values in relation to the non-rigid connection design may predispose biomechanical complications, such as ligament overstretching, cementum damage, and an increased risk of periodontal disease [68,69,70]. Previous studies suggested that excessive micromotion can lead to mechanical failures, including microfractures and wear of the prothesis supporting structures [71,72,73]. The reduced micromotion in rigid connectors likely contributes to better load distribution over the FPD, which is crucial for the longevity of the prosthesis and protection of the natural tooth abutment from intrusion, preserving the PDL [46,74,75,76,77]. Tsaousoglou et al. [66] reported no dental intrusion in tooth-implant fixed partial dentures with rigid connectors. Additionally, Ting et al. [78] further explained that the incidence of tooth intrusion was linked to the use of non-rigid connectors. This is particularly important in cases where long-term success and patient comfort are paramount.

Another important aspect is the combination effect of the non-rigid connection and the elasticity of the periodontal ligament system. After load application, the natural tooth is allowed free movement and becomes disproportionately displaced in relation to the dental implant, leaving the implant in an unfavorable position, bearing most of the loading forces [79]. It was suggested that such implant overload would eventually initiate peri-implant bone resorption, affect osseointegration, and ultimately cause implant failure with prosthetic problems [80]. In addition, the possible hyperfunction of the natural tooth would result in the atrophy of the periodontal structures. The persistent pressure on the periodontal ligament induces a series of biological responses well documented in orthodontic studies. The sequence of events is initiated when blood flow is compromised in the compressed PDL, followed by cell death in this area, commonly termed hyalinization [81]. Macrophages subsequently remove the hyalinized tissue, and bone degradation occurs due to osteoclast activity. These events together ultimately lead to the intrusion of the tooth [81,82,83]. Furthermore, the literature indicates strong correlation between non-rigid connection and the increase in complications and inter-review appointments required to address technical issues [84,85,86]. In a study by Nickenig et al. [86], only 3 out of 56 rigidly connected tooth-implant FPDs experienced technical complications, whereas 8 out of 28 non-rigidly connected tooth-implant FPDs required modifications.

Several authors suggest that a rigidly connected tooth-implant restoration unit still possesses sufficient flexibility to enable tooth movement within the socket, thereby allowing the tooth to contribute to support [87,88]. This results in a more balanced force distribution between the tooth and the implant [89]. The distribution of force may be attributed to the flexibility of the prosthesis and abutment screw, implant fixture micromovement within the bone, and the inherent flexibility of the rigid prosthesis. Clinical evidence supporting these claims of equal force distribution has been demonstrated through a strain-gauge analysis of loads applied to the prostheses [90].

Our findings also support this evidence, where Group A (rigid connection) displayed a degree of displacement and micromovement across all measured regions within the tooth-implant FPD (Table 1, Figure 9a–f, Figure 10a–f, Figure 11a–f and Figure 12a–f). This rationale could render using mobile elements in prostheses unnecessary, favoring rigid prostheses for connecting natural teeth to implants. Essentially, a vertical bite force causes the tooth to move within the periodontal ligament, generating a moment of force around the implant. However, the magnitude of this moment is influenced by the mobility of the tooth and implant, the length and flexibility of the prosthesis and its components, and the bone’s flexibility. If all elements within the implant-restoration unit possess sufficient mobility and the tooth remains stable within the socket, both the implant and tooth will contribute to supporting the fixed partial denture, ultimately averting long-term complications. Pesqueira et al.’s [91] in vitro study supported this claim by reporting that rigid connection designs exhibited a degree of micromovement, yet it was still significantly less under occlusal loading compared to non-rigid connectors.

The maximum displacement observed at the dentin present at the interface between the crown margin/tooth finish line in Group B (non-rigid) were significantly higher than Group A (*p* value = 0.043 *) (Table 2 and Table 3, Figure 9c–i, Figure 10c–i, Figure 11c–i and Figure 12c–i). Depending on the duration, distribution, and direction of occlusal loads, this could potentially lead to the debonding the natural tooth retainer, which can go undetected as the prothesis remains attached to the implant [92,93,94]. Bragger et al. [95] documented this phenomenon and reported the development of caries in the tooth abutment. Another interesting finding of this study was that the implant fixture demonstrated a degree of displacement after the application of load, although it was less pronounced than that of the natural tooth. There is a clear distinction in how the tooth and the implant respond to movement. For the tooth, the periodontal ligament (PDL) cushions the occlusal force, preventing it from reaching the bone and initiating the hyalinization process. In contrast, the bone directly absorbs forces from the implant, causing it to temporarily deform before returning to its original shape. Consequently, while tooth movement is clinically observable, implant movement is not readily detectable [88]. For the implant, the maximum displacement was present at the implant–abutment body in both groups. Although displacement values recorded in the Group B (non-rigid) were higher but did not display statistical significance, this can be attributed to the use of unidirectional vertical static load. Under vertical loading, there is a direct correlation between abutment displacement and implant apical displacement, meaning that the majority of the applied load could have the potential to cause bone deformation. Conversely, under oblique loading conditions, abutment displacement does not reflect implant displacement due to possible implant rotation [41]. However, Zhang et al. [96] reported that under simulated multiaxial dynamic chewing load, displacement occurred at the interface between the implant and abutment in all tested groups, with the extent of micromotion varying with varying load magnitudes. This resulted in a horizontal displacement of 0.075–1.459 μm and 0.091–0.945 μm, leading to palatal and distal microgaps in the implant–abutment interface. Gratton et al. [97] also verified that while the implant system may seem stable under typical observation, increased micro-fretting occurs at lower pre-tightening torques.

It is important to note that significant micromotion at the implant body may lead to the passage of fluid through the abutment–implant interface [97]. This fluid leakage has the potential to transmit bacteria or pathogens from the surrounding tissue into the abutment space [98]. Additionally, micromotion can create a “pumping” effect, allowing fluid to move in and out to the adjacent bone, potentially leading to crestal bone loss [99]. Although displacement values observed in the implant–abutment complex and tooth abutment margin were below the tolerance motion limit reported in the literature, these displacement values are subject to increase under dynamic multiaxial chewing loads, especially with the use of non-rigid connection design.

Furthermore, displacement values measured at the mandible demonstrated a notably significant increase (*p* value = 0.021 *) when a non-rigid connection was used (Table 3, Figure 4, Figure 9b–h, Figure 10b–h, Figure 11b–h and Figure 12b–h). When an implant is subjected to load, it is transmitted to the surrounding bone, creating compression on one side and tension on the other. This highlights the critical nature of both compressive and tensile strengths on trabecular bone. Various studies have offered different insights: one study found that both strengths are equivalent, other studies indicated that the compressive strength was superior, while another study revealed the presence of greater tensile strength [100,101,102]. As long as the strain remains within the elastic limit of the bone’s stress–strain curve, the trabecular bone will revert to its original form once the force is halted, allowing the implant to also return to its initial position. Therefore, bone quality is a crucial factor to consider as it affects the biomechanical behavior of the natural tooth to implant connection [103]. Although it is not feasible to clinically measure the extent of micromotion at the bone–implant interface during mastication, insights from this study highlights the importance of implementing all possible measures to prevent or reduce micromotion at the bone–implant interface especially in immediately loaded implants [104].

The non-rigid connection is intentionally designed to split the prosthesis into two sections: a single crown attached to the tooth, and a cantilever on the implant side that disrupts the continuity of the prosthesis and its material. This can explain the maximum displacement recorded at the FPD bridge at the connection between the tooth retainer and the pontic when a non-rigid connector was used (*p* value = 0.021 *) (Figure 9a–g, Figure 10a–g, Figure 11a–g and Figure 12a–g, Table 3). The elastic modulus of the periodontal ligament (PDL) is significantly lower than that of other supporting structures: approximately 2000 times lower than cortical bone, 200 times lower than trabecular bone, and 33.5 times lower than low-density trabecular bone. This disparity creates a complex biomechanical environment where occlusal forces, transmitted through the metal-ceramic alloy (elastic modulus around 110 GPa) to the titanium implant and tooth, ultimately affect the PDL and bone. The rigidity of the implant and tooth compared to the PDL and trabecular bone leads to distortion observed through their displacement. The tooth displaces apically, varying with the type of connection, whether rigid or non-rigid [38,105,106,107]. For illustration, the 4.7 mm diameter and 10 mm length implant displayed the highest difference in displacement, where the use of non-rigid connection design resulted in a 91.1-fold higher apical tooth displacement (0.81100 mm) than its corresponding rigid connection design (0.00890 mm) (Table 2, Figure 7).

A study by Huang et al. [108] demonstrated that while rigid connectors might offer better stability, they do not accommodate the differential movement between the natural tooth and the implant. As a result, they generate higher stresses in the cortical bone surrounding the implant, potentially leading to complications such as bone resorption or implant loosening over time. In another study focusing on connection design, Gowda et al. [109] compared three different fixed dental prosthesis (FDP) models that connected a mesial natural tooth to a distal implant. In the first model, both the mesial and distal connectors were rigid. The second model featured a mesial non-rigid connector and a distal rigid connector, while the third model had a mesial rigid connector and a distal non-rigid connector. The highest stress values attributed to the rigid–rigid connector design were generated in the crestal bone of the supporting structures, and it was recommended to use a non-rigid connector on the distal aspect of the pontic for stress reduction. Furthermore, Kanojia et al. [110] used a non-rigid connector for an FPD design with a pier abutment. The non-rigid connector allowed movement within the FPD, effectively redirecting stresses away from the tooth abutment. The authors concluded that connector design is crucial in pier abutment cases, as it plays a key role in determining the success of the FPD.

Materials with a higher modulus of elasticity exhibit greater stiffness, leading to reduced deformation under occlusal loads. Importantly, the high modulus not only results in lower immediate displacements but may also contribute to a slower rate of displacement over time. This gradual displacement response allows for more controlled stress distribution and adaptation by the surrounding bone and soft tissues, potentially enhancing the long-term stability and acceptance of the implant system. The slower displacement reduces the risk of micromovements at the implant–bone interface, which is crucial for maintaining osseointegration and minimizing complications such as bone resorption or implant overload. These findings align with previous studies that have highlighted the benefits of time-dependent displacement behavior in improving the biomechanical performance and clinical outcomes of dental implants [111,112,113]. In the context of prosthetic stability, slower displacement is advantageous because it helps in maintaining the correct positioning of the prosthesis over time. This is particularly important for tooth-implant fixed partial dentures, where differential movement between the tooth and implant could otherwise lead to mechanical failures.

The data suggest a negative correlation between implant length and displacement, where shorter implants tend to have lower displacement values. In terms of diameter, wider implants also exhibit lower displacement, indicating better stability. However, a combination of wide implant diameter with proportional implant length is necessary to maintain low displacement values (Table 4, Figure 13 and Figure 14). Among the combinations studied, the 5.7 mm × 10 mm in the non-rigid connector group showcased the best performance, exhibiting the least displacement values across most measured components (Figure 8). This implies that shorter and wider implants might offer more resistance to displacement forces, making them preferable in scenarios where minimizing micromotion is crucial [114,115,116]. This finding aligns with Naguib et al.’s [46] stress analysis, where the same combination of length and diameter used with a rigid connection design provided optimum biomechanical performance. A recent study by Hashemi et al. [117] also recommended against the use of a non-rigid connection design for the construction of tooth-implant FPDs in the posterior region.

This study has several limitations that may impact the generalizability of the results. Firstly, the experimental and computational models used idealized vertical forces, while normal occlusal forces during mastication include both axial and shear loads. Although axial loading constitutes most of the occlusal force during chewing [118], future studies should examine the response of dental implants and natural teeth to shear loads. Secondly, the laboratory assumption of full osseointegration for the implant may not accurately reflect the actual integration within natural bone. Additionally, the finite element models assumed homogeneous material properties for the mandible, despite the fact that cortical and trabecular bone have variable densities and exhibit inhomogeneous material behavior [119].

Finally, this investigation represents only the beginning of complex investigations that the authors intend to carry out in the coming period. However, clinicians should carefully evaluate the specific clinical scenario and patient needs when selecting the appropriate connector design and implant dimensions. Our findings coincided with this study’s hypothesis that rigid connectors are generally recommended for enhancing the biomechanical stability of tooth-implant fixed partial dentures.

## 5. Conclusions

This study provided a comprehensive analysis of the biomechanical performance of tooth-implant fixed partial dentures (FPDs) with a focus on the influence of connector design. The findings clearly demonstrate that the use of rigid connectors significantly reduces displacement and micromotion across various components of the FPD, including the bridge, mandible, dentin, cementum, and periodontal ligament (PDL). Notably, the rigid connector design exhibited superior biomechanical stability compared to non-rigid connectors, which is crucial for the long-term success of hybrid prostheses.

Among the different combinations of implant lengths and diameters analyzed, the 5.7 mm × 10 mm implant in the rigid connector group showed the best overall performance, in relation to recorded displacement and micromotion. This suggests that the combination of a wider diameter and proportional implant length can enhance the stability and longevity of the prosthesis.

Clinically, these findings support the recommendation of using rigid connectors in tooth-implant FPDs, particularly in cases where minimizing micromotion is critical to avoid complications such as ligament overstretching, cementum damage, and implant overload. These findings contribute to the ongoing development of more effective and durable dental prostheses, with the potential to improve patient outcomes in the context of hybrid tooth-implant restorations.

Future research should explore the effects of dynamic and oblique loading on the biomechanical behavior of tooth-implant FPDs to provide a more comprehensive understanding of their performance in various clinical scenarios.

## Figures and Tables

**Table 1:** Material properties assigned to implant materials, dental tissues, prosthesis, and bone.

Materials	Modulus of Elasticity (MPa) × 10^6^	Poisson’s Ratio
Titanium implant	110,000 [33,48]	0.35 [33,48]
Cortical bone	15,000 [33,49]	0.30 [33,49]
Cancellous bone	1500 [33,49]	0.30 [33,49]
Dentin	18,600 [50]	0.31 [50]
Cementum	16,000 [46]	0.3 [46]
Periodontal ligament	69 [48,51]	0.45 [48,51]
Nickel–chromium	185,000 [52]	0.30 [52]

**Table 2:** Displacement in tooth-implant FPD with different designs measured in different components.

	FPD Bridge	Mandible	Dentin	Cementum	PDL	Implant
Group A						
3.7–11.5 mm	0.0086380	0.00205	0.00819	0.00816	0.00774	0.0041430
3.7–13 mm	0.0066230	0.00178	0.00625	0.00623	0.00611	0.0032770
4.7–10 mm	0.0103700	0.00162	0.00983	0.00976	0.00890	0.0045110
5.7–10 mm	0.0079330	0.00198	0.00762	0.00759	0.00682	0.0034650
Group B						
3.7–11.5 mm	0.6640000	0.17150	0.63620	0.63300	0.63270	0.295800
3.7–13 mm	0.0142900	0.00226	0.01022	0.01018	0.00923	0.0058260
4.7–10 mm	0.9543000	0.15310	0.90940	0.90290	0.81100	0.388700
5.7–10 mm	0.0120300	0.00215	0.00846	0.00841	0.00778	0.0040610

**Table 3:** Comparison between displacement values measured in Group A (rigid) and Group B (non-rigid) tooth-implant FPDs designs at different components.

Displacement Points	Group A (Rigid Connection)	Group B (Non-Rigid Connection)	Significance
Bridge	0.0084 ± 0.002	0.4112 ± 0.237	*p* value = 0.021 *
Percentage changes (%)	-	4795.24%	
Mandible	0.0019 ± 0.0002	0.39107 ± 0.455	*p* value = 0.021 *
Percentage changes (%)	-	20482.63%	
Dentin	0.0080 ± 0.0015	0.39107 ± 0.455	*p* value = 0.043 *
Percentage changes (%)	-	4788.38%	
Cementum	0.0079 ± 0.0014	0.38862 ± 0.4517	*p* value = 0.043 *
Percentage changes (%)	-	4819.24%	
PDL	0.0074 ± 0.0012	0.36518 ± 0.4182	*p* value = 0.043 *
Percentage changes (%)	-	4834.86%	
Distal implant	0.0038 ± 0.0006	0.17360 ± 0.1984	*p* value = 0.083
Percentage changes (%)	-	4468.42%	

Data expressed as mean ± standard deviation. * Indicates statistical significance (* *p* < 0.05) between Group A (rigid connection) and Group B (non-rigid connection), as determined by the Mann-Whitney test. Percentage changes were determined by the following equation displacement in Group B—displacement on Group A/displacement in Group A × 100.

**Table 4:** Correlations between implant diameter and length and different displacement.

Variable	Implant Diameter		Implant Length	
	Correlation Coefficient (r)	*p*-Value	Correlation Coefficient (r)	*p*-Value
Displacement induced in Bridge	−0.180	0.670	−0.259	0.535
Displacement induced in Mandible	0.026	0.952	−0.185	0.660
Displacement induced in Dentin	−0.180	0.670	−0.309	0.457
Displacement induced in Cementum	−0.180	0.670	−0.309	0.457
Displacement induced in PDL	−0.180	0.670	−0.309	0.457
Displacement induced in Distal implant	−0.103	0.808	−0.272	0.515

## Data Availability

The original contributions presented in the study are included in the article, further inquiries can be directed to the corresponding author.

## Abbreviations

FPD	Fixed partial dentures
PDL	Periodontal ligament
CT	Computed tomography
ROI	Region of interest
DICOM	Digital Imaging and Communications in Medicine
Ni-Cr	Nickel–chromium
TRIPOD	Transparent reporting of a multivariable prediction model for Individual Prognosis or Diagnosis
FDP	Fixed dental prosthesis
*p*-value “*p*”	Probability value of significance
